# Antipyretic Therapy in Critically Ill Patients with Sepsis: An Interaction with Body Temperature

**DOI:** 10.1371/journal.pone.0121919

**Published:** 2015-03-30

**Authors:** Zhongheng Zhang, Lin Chen, Hongying Ni

**Affiliations:** Department of Critical Care Medicine, Jinhua Municipal Central Hospital, Jinhua Hospital of Zhejiang University, Zhejiang, P. R. China; Hospital Sirio-Libanes, BRAZIL

## Abstract

**Background and Objective:**

The effect of antipyretic therapy on mortality in patients with sepsis remains undetermined. The present study aimed to investigate the role of antipyretic therapy in ICU patients with sepsis by using a large clinical database.

**Methods:**

The multiparameter intelligent monitoring in intensive care II (MIMIC- II) database was employed for the study. Adult patients with sepsis were included for analysis. Antipyretic therapy included antipyretic medication and external cooling. Multivariable model with interaction terms were employed to explore the association of antipyretic therapy and mortality risk.

**Main Results:**

A total of 15,268 patients fulfilled inclusion criteria and were included in the study. In multivariable model by treating temperature as a continuous variable, there was significant interaction between antipyretic therapy and the maximum temperature (T_max_). While antipyretic therapy had no significant effect on mortality in low temperature quintiles, antipyretic therapy was associated with increased risk of death in the quintile with body temperature >39°C (OR: 1.29, 95% CI: 1.04–1.61).

**Conclusion:**

Our study shows that there is no beneficial effect on reducing mortality risk with the use of antipyretic therapy in ICU patients with sepsis. External cooling may even be harmful in patients with sepsis.

## Introduction

Sepsis is among the most important causes of morbidity and mortality in the intensive care unit. It is estimated that about 5% deaths are directly attributable to sepsis [1]. Depending on different severities of illness varying from sepsis, severe sepsis and septic shock, patients with sepsis show mortality rates ranging from 10% to 40% [2,3]. Diagnostic criteria of sepsis are easy to fulfill, requiring only the confirmation of systematic inflammatory response syndrome (SIRS) and documented or suspected infection [4]. Because sepsis continues to be a great threat to human health, the surviving sepsis campaign has made tremendous efforts to reduce its morbidity and mortality. Strategies such as early goal directed therapy, low tidal volume ventilation, early use of appropriate antibiotics have shown some beneficial effects on sepsis patients. However, the improvement is only minimal and the mortality rate of sepsis remains high. Therefore, new studies begin to focus on other novel medications or strategies for the improvement of medical care for patients with sepsis [5].

Fever is the cardinal symptom of sepsis, and is the most important reason for hospital visit. Most physicians will prescribe antipyretic therapy to patients with sepsis, aiming to relieve the symptom of fever. They believe that fever increases metabolic rate via sympathetic activation, making the imbalance between oxygen demand and supply even more severe [6]. This is particularly true in patients with septic shock requiring extracorporeal life support. In this regard, antipyretic therapy is thought to be beneficial for sepsis patients. However, opponents contend that fever is a natural response of human body to microorganism infection and may help to inhibit the growth of microorganism. Therefore, they usually withhold antipyretics when fever is caused by infection, as is the case in sepsis [7–9].

Investigations on antipyretic therapy have never waned from the very beginning of 1990s [10–13]. Both observational studies and randomized controlled trials (RCT) have been conducted but their results are conflicting. The problem is attributable to variations in study populations, designs, and protocols for performing antipyretic therapy. For instance, body temperature is a continuous variable and the effect of antipyretic therapy on mortality may be altered by different levels body temperature. In other words, there could be an interaction between body temperature and mortality. However, the inclusion of interaction terms substantially increases the degree of freedom in the multivariable model. For most of previous studies, this could not be done due to small sample size (e.g. the sample size cannot support too many degrees of freedom, otherwise, the problem of overfitting arises). The present study utilized a large clinical database to allow for models with complexity. Fractional polynomials and interaction terms were explored after the main effect model was determined. We hypothesized that the effect of antipyretic therapy on mortality would be modified by body temperature.

## Materials and Methods

### Clinical database

The multiparameter intelligent monitoring in intensive care II (MIMIC- II) database was employed for the study. MIMIC- II was a freely available database comprising more than 30,000 ICU patients. Patients’ information on demographics, laboratory findings, imaging study, vital signs and progress notes were available [14]. The institutional review boards of the Massachusetts Institute of Technology (Cambridge, MA) and Beth Israel Deaconess Medical Center (Boston, MA) approved the establishment of the database. De-identification was performed to ensure patients’ confidentiality. Our access to the database was approved after completion of the national institute of health (NIH) web-based training course named “Protecting Human Research Participants” by the author Z.Z. (certification number: 1132877).

### Subject selection

Adult patients meeting the criteria of sepsis were included for analysis. The diagnosis of sepsis was adapted from that defined in Surviving sepsis Campaign [4]. SIRS was defined as fulfilling two or more of the following criteria within 24 hours after ICU admission: 1) fever (>38.3°C) or hypothermia (<36°C); 2) tachycardia (>90/min); 3) leukocytosis (WBC count>12000/μL) or leukopenia (WBC count<4000/μL); 4) tachypnea (>20/min). If there were multiple measurements within 24 hours, the one most likely to meet the criteria was adopted (e.g. highest or lowest temperature, highest heart rate). Infection was defined as documented or suspected. Infection was defined if one of the following criteria was fulfilled 1) ICD9 contains the term “infection” or “pneumonia”; 2) microbiological culture was positive. Data management was performed by using the software Stata 13.1 (College Station, Texas 77845 USA). The code for the selection of sepsis patients is shown in supplemental file (S1 Appendix).

### Data extraction

Antipyretic therapy consisted of antipyretic medication and external cooling. The former included drugs such as acetaminophen, ibuprofen, naproxen, valtaren, ketoprofen, nimesulide, diclofenac. The latter included blanket cooling and ice pack. Structural query language (SQL) to extract external cooling was:

$$\begin{array}{l} select\;*\;from\;chartevents\\ where\;itemid=134\;OR\;itemid=7281 \end{array}$$

All measurements of body temperature recorded during ICU stay were extracted. A total of 1,164,474 measurements were obtained. Other variables including initial lactate [15], sequential organ failure assessment (SOFA), simplified acute physiology score (SAPSⅠ), age at ICU admission, gender and ICU type were extracted. There were four types of ICU: medical ICU (MICU), SICU (surgical ICU), coronary care unit (CCU) and cardiac surgery recovery unit (CSRU).

ICU mortality was used as the study endpoint. It was defined as the status of a subject at ICU discharge (dead vs. alive). It was a solid outcome that was not subject to bias.

### Definitions of body temperature

**Fig 1:**
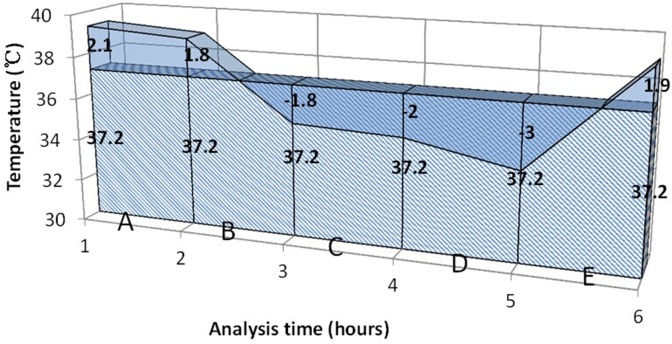
Definition of temperature load. The shaded area indicates normal temperature. The blue non-shaded area is temperature exceeding 37.2°C, termed temperature load.

Because temperatures were measured repeatedly for each subject, some definitions of temperature were adopted to accommodate the magnitude and duration of temperature. The measurement of temperature recorded in the database was not standardized but could be measured via rectum, oral cavity, axilla or tympanic membrane. The measurement site was not recorded in the database. Temperature load was defined as the difference between actual temperature area and normal temperature area (Fig. 1). We acknowledged that there were circadian changes in body temperature and it was difficult to simply define a cutoff point [16–18]. However, we defined fever as body temperature>37.2°C, which was recommended in the classic medical textbook Harrison’s internal medicine [19]. Temperature values at each measurement time were connected with the time as the horizontal axis. Actual temperature area was the area under the actual temperature line, and normal temperature area was the area under normal temperature (37.2°C). If the temperature load was less than or equal to zero, it was defined as no temperature load (coded as 0 for the variable). The calculation was performed for each time where temperature was measured. Maximum temperature (T_max_) was defined as the highest temperature during ICU stay.

### Missing data management

Variables with missing data were common in the MIMIC-Ⅱ database. If the percentage of missing observations was less than 5%, we replace the missing variable with mode or mean value of that variable. If a variable had more than 30% observations missing, we used dummy variable to recode the missing observations. In the dataset, we found that 40% of the lactate values were missing. We coded missing lactate value as 2, and assigned 0 and 1 for normal lactate and hyperlactatemia, respectively. If missing values accounted for 5%–30%, multiple imputation technique was performed for further estimation [20].

### Statistical analysis

Variables were expressed as mean±standard deviation (SD) or median and 95% confidence interval (CI) as appropriate. Bivariate analysis was performed to compare the difference between survivors and non-survivors.

Multivariable regression model was established by treating temperature as continuous variable. Both SASP-I and SOFA represented the severity of illness and only SOFA was included in the model. Another reason by doing so was that SOFA did not incorporate age as a component. Since we had already included age in the model, incorporation of scores with age may increase the risk of collinearity. All other covariates were included into the multivariable regression model. The model incorporated the maximum body temperature (T_max_) as a continuous variable. Fractional polynomials method was employed to explore the linearity of continuous variables such as age and T_max_ [21]. Closed test procedure was used to examine whether the model with higher power terms performed better than the linear model. If there was no statistical significance at p = 0.05 level, the linear model was adopted for the principal of parsimony. Interactions between antipyretic therapy and body temperature were explored to examine whether the effect of antipyretic therapy on mortality differs at different levels of body temperature. If there was statistically significant interaction, odds ratios of antipyretic therapy would be reported at different levels of body temperature.

All statistical analyses were performed by using Stata 13.1 (College Station, Texas 77845 USA). Statistical significance was considered at p<0.05.

## Results

A total of 32,319 adult ICU patients were identified in the MIMIC-Ⅱ database, including 15,268 patients meeting the criteria of sepsis. There were significant differences in many variables between survivors and non-survivors (Table 1). Survivors were younger than non-survivors (64.0±20.4 vs 69.6±16.2 years, p<0.001). Patients with hyperlactatemia were more likely to die than those with normal or missing values (p<0.001). Patients in MICU were associated with increased risk of death (50.98% vs. 42.96%, p<0.001), whereas those in CSRU were less likely to die (20.92% vs. 29.73%, p<0.001). As expected, non-survivors showed significantly higher SOFA (9.95±4.51 vs. 5.88±3.77, p<0.001) and SAPS-Ⅰ(19.21±5.54 vs. 14.11±5.12, p<0.001) scores. Fever was associated with increased risk of death, irrespective of how it was measured (within 24 hours after ICU admission, during ICU stay and temperature load). Antipyretic therapy was associated with increased risk of death (17.71% vs. 11.49%, p<0.001). However, there was no effect of the use of antipyretic medications (6.63% vs. 7.46%, p = 0.198).

**Table 1:** Comparison of clinical characteristics between ICU survivors and non-survivors.

Variables	Overall (n = 15268)	Survivors (n = 13538)	Non-survivors (n = 1730)	p
Age (years)	64.6±20.0	64.0±20.4	69.6±16.2	<0.001
Gender (male vs)	8175 (53.54)	7240 (53.48)	935 (54.05)	0.656
Lactate				<0.001
Normal (<2mmol/l)	4842 (31.71)	4246 (31.36)	596 (34.45)	
Hyperlactamia (>2 mmol/l)	4336 (28.40)	3429 (25.33)	907 (52.43)	
Missing values	6090 (39.89)	5863 (43.31)	227 (13.12)	
Care unit				<0.001
MICU	6,698 (43.87)	5,816 (42.96)	882 (50.98)	
SICU	1,050 (6.88)	973 (7.19)	77 (4.45)	
CCU	3,133 (20.52)	2,724 (20.12)	409 (23.64)	
CSRU	4,387 (28.73)	4,025 (29.73)	362 (20.92)	
SOFA	6.34±4.07	5.88±3.77	9.95±4.51	<0.001
SAPS-Ⅰ	14.67±5.41	14.11±5.12	19.21±5.54	<0.001
Fever in 24 hours	9287 (60.83)	8028 (59.30)	1259 (72.77)	<0.001
Fever during ICU stay	11433 (74.88)	10036 (74.13)	1397 (80.75)	<0.001
Temperature load (°C×hr)	32.01 (30.77, 33.26)	30.26 (28.95, 31.56)	45.75 (41.79, 49.70)	<0.001
Antipyretic therapy of any method	1852 (12.13)	1555 (11.49)	297 (17.17)	<0.001
Antipyretic medication	1027 (6.73)	898 (6.63)	129 (7.46)	0.198
External cooling	1006 (6.59)	794 (5.86)	212 (12.25)	<0.001

Abbreviations: MICU, medical intensive care unit; SICU, surgical intensive care unit; CCU, coronary care unit; CSRU, Cardiac Surgery Recovery Unit

**Fig 2:**
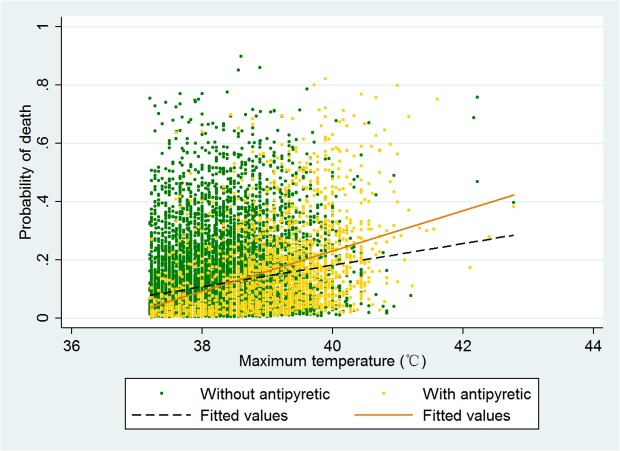
The relationship between probability of death and T_max_. The Probability of death increased with increasing T_max_, and the slope was altered by the use of antipyretic therapy.

In the regression model T_max_ was incorporated into the model as a continuous variable (Table 2). There was significant interaction between T_max_ and antipyretic therapy. When there was no antipyretic therapy (coded as 0), T_max_ was significantly associated with mortality risk (OR: 1.15, 95% CI: 1.07–1.23). The mortality risk slope was steeper in antipyretic therapy group than non-antipyretic group (Fig. 2). We further categorized temperature into quintiles and the result showed that the effect of antipyretic therapy had no significant effect on mortality in the four lower quintiles, while antipyretic therapy was associated with increased risk of death in the quintile with body temperature>39°C (OR: 1.29, 95% CI: 1.04–1.61, Table 3). When temperature load was incorporated into the model, antipyretic therapy remained to have adverse effect on mortality (OR: 1.355, 95% CI: 1.153–1.593). However, temperature load was not associated with mortality outcome in this model (Table 4).

**Table 2:** Adjustment for the effect of antipyretic therapy for patients with sepsis by treating temperature as continuous variable (maximum body temperature during ICU stay).

Odds Ratio	Odds ratio	Lower limit of 95% CI	Upper limit of 95% CI	P
Age	1.02	1.01	1.02	<0.001
SOFA	1.22	1.20	1.24	<0.001
Sex	1.12	1.003	1.251	0.044
Care unit (MICU as the reference)				
SICU	0.70	0.54	0.90	0.006
CCU	0.96	0.83	1.10	0.524
CSRU	0.46	0.40	0.53	<0.001
Lactate (normal as the reference)				
Hyperlactamia (>2 mmol/l)	1.30	1.15	1.48	<0.001
Missing values	0.47	0.40	0.56	<0.001
Maximum temperature during ICU stay (with each 1°C increase) ¶	1.15	1.07	1.23	<0.001
Antipyretic therapy	.0000164	1.98e-08	.013631	<0.001
Antipyretic therapy×T_max_ ‡	1.33	1.12	1.58	<0.001

¶ The main effect of T_max_ (OR = 1.15) is exponentiation of the slope in the regression model when no antipyretic therapy was given (antipyretic therapy = 1).

‡ The coefficient of the interaction term was the difference of the slope in groups with and without antipyretic therapy. An OR>1 indicates a positive coefficient and steeper slope of the antipyretic therapy group than non-antipyretic group.

**Table 3:** Effect of antipyretic therapy on mortality (expressed as odds ratio) at different degrees of body temperature.

Temperature range (°C)	Odds ratio¶	95% CI	P
<37.2	1.65	0.77–3.53	0.196
37.2–37.7	0.98	0.50–1.90	0.950
37.7–38.2	0.97	0.52–1.82	0.930
38.2–39	0.73	0.51–1.04	0.082
>39	1.29	1.04–1.61	0.020

¶The odds ratios were obtained by fitting the main effect model without interaction terms.

**Table 4:** Adjustment for the effect of antipyretic therapy for patients with sepsis by using temperature load as continuous variable.

	Odds ratio	Lower limit of 95% CI	Upper limit of 95% CI	P
Age	1.016	1.013	1.019	<0.001
SOFA	1.222	1.205	1.240	<0.001
Sex	1.098	0.984	1.226	0.095
Care unit (MICU as the reference)				
SICU	0.686	0.530	0.888	0.004
CCU	0.963	0.839	1.105	0.590
CSRU	0.458	0.399	0.527	<0.001
Lactate (normal as the reference)				
Hyperlactamia (>2 mmol/l)	1.303	1.151	1.474	<0.001
Missing values	0.450	0.381	0.531	<0.001
Antipyretic therapy	1.355	1.153	1.593	<0.001
Temperature load	1.000	0.999	1.001	0.904
Constant term	0.011	0.008	0.014	<0.001

Abbreviations: MICU, medical intensive care unit; SOFA: sequential organ failure assessment; SICU, surgical intensive care unit; CCU, coronary care unit; CSRU, Cardiac Surgery Recovery Unit.

**Fig 3:**
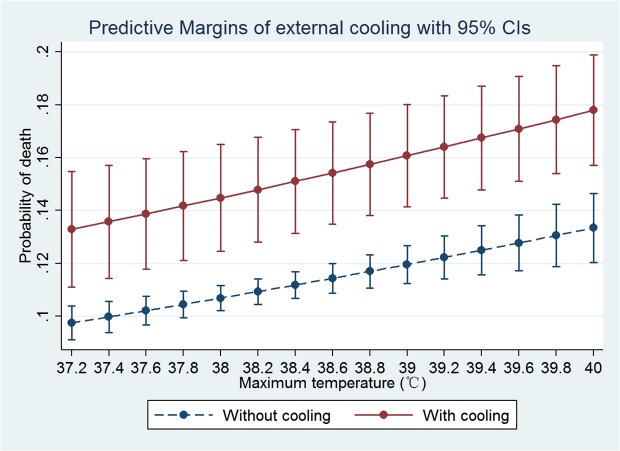
Probability of death increased with increasing T_max_. Use of external cooling significantly increased the risk of death, but there was no interaction between external cooling and T_max_.

Furthermore, we restricted to external cooling in multivariable analysis (Table 5). Again there was no fractional polynomial term or interactions. External cooling was associated with increased risk of death (OR: 1.51, 95% CI: 1.23–1.84). The predictive margins of patients with and without external cooling are shown in Fig. 3. Some patients received both external cooling and antipyretic mediations. To exclude the influence of antipyretic mediations, we performed sensitivity analysis by restricting to those without antipyretic mediations and the model was refitted. There were 14,241 observations included into the model and the result showed that external cooling was still an independent risk factor for mortality risk (OR: 1.40, 95% CI: 1.12–1.74).

**Table 5:** Adjustment for the effect of external cooling for patients with sepsis by treating temperature as continuous variable (maximum body temperature during ICU stay).

	Odds Ratio	Lower limit of 95% CI	Upper limit of 95% CI	P>z
Maximum temperature (with each 1 degree increase)	1.16	1.09	1.24	<0.001
External cooling	1.51	1.23	1.84	<0.001
Age	1.02	1.01	1.02	<0.001
SOFA	1.22	1.20	1.24	<0.001
Sex	1.12	1.01	1.25	0.040
Care unit (MICU as the reference)				
SICU	0.66	0.51	0.86	0.002
CCU	0.95	0.83	1.09	0.498
CSRU	0.46	0.40	0.53	<0.001
Lactate (normal as the reference)				
Hyperlactamia (>2 mmol/l)	1.31	1.15	1.48	<0.001
Missing values	0.48	0.40	0.56	<0.001

The model contains no interaction terms.

## Discussion

The study showed that antipyretic therapy, especially the external cooling, had adverse impact on mortality outcome in ICU patients with sepsis. The adverse effect was independent of the body temperature. When T_max_ was incorporated into the model as continuous variable, there was significant interaction between body temperature and antipyretic therapy. In other words, the effect of antipyretic therapy on mortality risk could be modified by T_max_. We further explored the effect of antipyretic therapy on mortality in quintiles of T_max_ and found that while the effect was not statistically significant in the quintile with T_max_<39°C, the adverse effect was significant at the quintile with T_max_>39°C. Interestingly, the adverse effect of antipyretic therapy was only true for external cooling, and the use of antipyretic medication showed neutral effect.

Our finding supports the notion that elevated body temperature is a natural response to infection and it is beneficial to sepsis patients. The plausible reasons can be due to increased production of protective heat shock proteins, direct inhibition of microorganism growth, enhancement of antibiotic effectiveness and augmentation of immune function [8]. Although the adverse effects of hyperthermia such as increase in metabolic burden and oxygen consumption do exist, these effects may be outweighed by the beneficial effect in sepsis. For example, increased oxygen consumption may well be balanced by increasing oxygen supply in ICU. Several observational studies demonstrated that fever may confer protection against adverse outcome. In a study involving 612 patients with confirmed gram-negative bacteria, fever with 24 hours was shown to be protective against mortality risk [22]. In another study involving invasive candida infection, Leroy O and coworkers found that a body temperature >38.2°C at the onset of infection was an independent predictor of survival [23]. However, Laupland KB and colleagues[24] showed that fever was associated with increased mortality (20.3% vs. 12%, p < 0.0001), which was consistent with the result of the bivariate analysis in our study. The result was confirmed in multivariable regression model. Laupland’s study incorporated unselected ICU patients including those with trauma and brain injury, which may partly explain the difference. Several randomized controlled trails have been performed to explore the effect of antipyretic therapy on mortality. The results are conflicting. Only one study reported beneficial effect of antipyretic therapy on mortality risk [25], others reported either neutral or adverse effect [12,26–28]. These RCTs are of limited sample size, which are subject to sampling error.

A novel finding in our study was that the effect of antipyretic therapy on mortality could be modified by T_max_. To further explore the interaction between antipyretic therapy and T_max_, we categorized T_max_ into quintiles and found that the adverse effect of antipyretic therapy was only significant in the quintile with body temperature>39°C. It is probable that the beneficial effects of fever increase positively with temperature and only at relatively high temperatures the benefits outweigh the adverse effect of fever. Experimental studies have demonstrated that some protective heat shock proteins are produced at highest rate at high temperatures [29]. For instance, detectable Hsp70 protein expression required 24 h exposure at 38.5°C, 6h exposure at 39.5°C, and only 1h exposure at 41°C. These results support our finding that antipyretic therapy at higher temperature confers more adverse effects [30]. At lower temperature range, the beneficial effect may not be prominent and can be abolished by its adverse effect.

Of note, our study showed that the adverse effect of antipyretic therapy was limited to external physical cooling, and there was no significant difference in the proportion of patients using antipyretic medicines between survivors and non-survivors. External cooling acts by lowering skin temperature considerably more than core temperature, resulting in cutaneous vasoconstriction and increase in blood pressure. Furthermore, external cooling usually leads to muscular shivering thereby increasing metabolic rate, energy expenditure and oxygen consumption. These effects act in concert to counteract the metabolic benefit of antipyretic therapy [31]. As a result, external cooling appears to have much greater adverse effect on clinical outcomes than drugs as shown in our study.

The study is retrospective in nature and bears some inherent limitations. First, Antipyretic medications may also be used for pain control. The study was based on data mining of electronic medical record and it was difficult to determine the reason why antipyretic medications were prescribed in most cases. However, we believed that the most important reason for the use of antipyretic medications were to control fever in sepsis patients. Second, the clinical outcome was short-term mortality and it was largely unknown whether antipyretic therapy could improve other long-term outcomes. This point should be kept in mind in interpreting our findings. Third, the anatomic source of infection was not included in our analysis because it was technically impossible to determine the source of infection. Our study was based on data mining of a critical care big data. Since the source of infection has been shown to be an important determinant of prognosis [32], it could be a confounding factor and our result should be interpreted with caution.

In conclusion, our study shows that there is no beneficial effect on reducing mortality risk with the use of antipyretic therapy in ICU patients with sepsis. External cooling may even be harmful. However, the findings in the study are hypothesis generating at best due to above-mentioned limitations. In order to assure future relevance of the results of this study, prospective studies must be conducted to examine the effect of antipyretic therapy on mortality in sepsis patients.

## Supporting Information

S1 Appendix Stata code for extracting patients with diagnosis of sepsis. (DOCX)
