# Supplementary material for: Antipyretic Therapy in Critically Ill Patients with Sepsis: An Interaction with Body Temperature
Source: PLoS One. 2015 Mar 30;10(3):e0121919. doi: 10.1371/journal.pone.0121919 (PMC4378844; doi:10.1371/journal.pone.0121919)
Supplement: S1 Appendix — (DOCX) [file pone.0121919.s001.docx]

cd "D:\medicine\paper 2014\sepsis and antipyretics\data process"

capture log close

log using antipysep_data01_24hr, replace text

// antipysep_data01_24hr.do: generate heart rate for the first 24 hours after ICU entry

// zhongheng zhang 12August 2014

version 13.1

clear all

macro drop _all

set linesize 80

use "D:\medicine\paper 2014\sepsis and antipyretics\data process\icustay_event.dta", clear

drop if icustay_age_group=="middle"

keep icustay_id icustay_intime

codebook icustay_id

merge 1:m icustay_id using "D:\medicine\paper 2014\sepsis and antipyretics\data process\chartevents_hr.dta", keep(match)

generate double hr_time = clock( charttime , "YMDhms")

keep if hr_time>= icustay_intime & hr_time<=icustay_intime+24*60*60*1000

g hr_tag=1

drop icustay_intime charttime value1num _merge hr_time

bysort icustay_id: drop if icustay_id==icustay_id[_n-1]

save antipysep_data01_24hr.dta, replace

log close

exit

cd "D:\medicine\paper 2014\sepsis and antipyretics\data process"

capture log close

log using antipysep_data02_24rr, replace text

// antipysep_data02_24rr.do: generate respiratory rate for the first 24 hours after ICU entry

// zhongheng zhang 16August 2014

version 13.1

clear all

macro drop _all

set linesize 80

use "D:\medicine\paper 2014\sepsis and antipyretics\data process\icustay_event.dta", clear

drop if icustay_age_group=="middle"

keep icustay_id icustay_intime

codebook icustay_id

merge 1:m icustay_id using "D:\medicine\paper 2014\sepsis and antipyretics\data process\chartevents_rr.dta", keep(match)

generate double rr_time = clock( charttime , "YMDhms")

keep if rr_time>= icustay_intime & rr_time<=icustay_intime+24*60*60*1000

g rr_tag=1

drop icustay_intime charttime value1num _merge rr_time

bysort icustay_id: drop if icustay_id==icustay_id[_n-1]

save antipysep_data02_24rr.dta, replace

log close

exit

cd "D:\medicine\paper 2014\sepsis and antipyretics\data process"

capture log close

log using antipysep_data03_24temp, replace text

// antipysep_data03_24temp.do: generate temperature for the first 24 hours after ICU entry

// zhongheng zhang 16August 2014

version 13.1

clear all

macro drop _all

set linesize 80

use "D:\medicine\paper 2014\sepsis and antipyretics\data process\icustay_event.dta", clear

drop if icustay_age_group=="middle"

keep icustay_id icustay_intime

codebook icustay_id

merge 1:m icustay_id using "D:\medicine\paper 2014\sepsis and antipyretics\data process\chartevents_temp.dta", keep(match)

keep if charttime>= icustay_intime & charttime<=icustay_intime+24*60*60*1000

g temp_tag=1

drop icustay_intime charttime value1num _merge

bysort icustay_id: drop if icustay_id==icustay_id[_n-1]

save antipysep_data03_24temp.dta, replace

log close

exit

cd "D:\medicine\paper 2014\sepsis and antipyretics\data process"

capture log close

log using antipysep_data04_wbc, replace text

// antipysep_data04_wbc.do: generate wbc for the first 24 hours after ICU entry

// zhongheng zhang 16August 2014

version 13.1

clear all

macro drop _all

set linesize 80

import delimited "D:\medicine\paper 2014\sepsis and antipyretics\data process\labevents_wbc.csv", delimiter(";") varnames(1)

drop subject_id hadm_id itemid value flag valueuom

keep if valuenum>12 |valuenum<4

drop if icustay_id==.

save labevents_wbc.dta, replace

use "D:\medicine\paper 2014\sepsis and antipyretics\data process\icustay_event.dta", clear

drop if icustay_age_group=="middle"

keep icustay_id icustay_intime

codebook icustay_id

merge 1:m icustay_id using "D:\medicine\paper 2014\sepsis and antipyretics\data process\labevents_wbc.dta", keep(match)

generate double wbc_time = clock( charttime , "YMDhms")

keep if wbc_time>= icustay_intime & wbc_time<=icustay_intime+24*60*60*1000

g wbc_tag=1

drop icustay_intime charttime wbc_time valuenum _merge

bysort icustay_id: drop if icustay_id==icustay_id[_n-1]

save antipysep_data04_wbc.dta, replace

log close

exit

cd "D:\medicine\paper 2014\sepsis and antipyretics\data process"

capture log close

log using antipysep_data05_sirs, replace text

// antipysep_data05_sirs.do: generate patients with SIRS

// zhongheng zhang 16August 2014

version 13.1

clear all

macro drop _all

set linesize 80

use "D:\medicine\paper 2014\sepsis and antipyretics\data process\icustay_event.dta", clear

drop if icustay_age_group=="middle"

merge 1:1 icustay_id using "D:\medicine\paper 2014\sepsis and antipyretics\data process\antipysep_data01_24hr.dta", nogenerate

merge 1:1 icustay_id using "D:\medicine\paper 2014\sepsis and antipyretics\data process\antipysep_data02_24rr.dta", nogenerate

merge 1:1 icustay_id using "D:\medicine\paper 2014\sepsis and antipyretics\data process\antipysep_data03_24temp.dta", nogenerate

merge 1:1 icustay_id using "D:\medicine\paper 2014\sepsis and antipyretics\data process\antipysep_data04_wbc.dta", nogenerate

egen float sirs_score = rowtotal(hr_tag rr_tag wbc_tag temp_tag)

tabulate sirs_score

save antipysep_data05_sirs.dta, replace

log close

exit

cd "D:\medicine\paper 2014\sepsis and antipyretics\data process"

capture log close

log using antipysep_data06_ICDinfect, replace text

// antipysep_data06_ICDinfect.do: infection or pneumonia in ICD

// zhongheng zhang 17August 2014

version 13.1

clear all

macro drop _all

set linesize 80

import delimited "D:\medicine\paper 2014\sepsis and antipyretics\data process\icd_infection.csv", delimiter(";")

drop if hadm_id==.

contract hadm_id

g ICD_infect_tag=1

drop _freq

save antipysep_data06a_condense.dta, replace

use "D:\medicine\paper 2014\sepsis and antipyretics\data process\icustay_event.dta", clear

drop if icustay_age_group=="middle"

keep icustay_id icustay_intime hadm_id

codebook icustay_id hadm_id

merge m:1 hadm_id using "D:\medicine\paper 2014\sepsis and antipyretics\data process\antipysep_data06a_condense.dta",keep(match) nogenerate

drop hadm_id icustay_intime

save antipysep_data06_ICDinfect.dta, replace

log close

exit

cd "D:\medicine\paper 2014\sepsis and antipyretics\data process"

capture log close

log using antipysep_data07_microbiology, replace text

// antipysep_data07_microbiology.do: generate microbiology result ICU entry

// zhongheng zhang 17August 2014

version 13.1

clear all

macro drop _all

set linesize 80

//generate icu stay for each subject in wide format

use "D:\medicine\paper 2014\sepsis and antipyretics\data process\icustay_event.dta", clear

keep subject_id icustay_id icustay_intime icustay_outtime

by subject_id, sort : egen float icu_seq = seq()

drop icustay_id

reshape wide icustay_intime icustay_outtime, i(subject_id) j(icu_seq)

save antipysep_data07a_icuforeachsubject.dta, replace

//match to microbiological data on suject id

import delimited "D:\medicine\paper 2014\sepsis and antipyretics\data process\microbiology.csv", delimiter(";") clear

merge m:1 subject_id using "D:\medicine\paper 2014\sepsis and antipyretics\data process\antipysep_data07a_icuforeachsubject.dta", keep(match) nogenerate

generate double microbiology_time = clock( charttime , "YMDhms")

drop if microbiology_time==.

forvalues i=1/47 {

g tag`i'=1 if microbiology_time>=icustay_intime`i' & microbiology_time<=icustay_outtime`i'

}

egen float total = rowtotal(tag1- tag47)

drop if total==0

keep subject_id tag1-tag47

contract subject_id- tag47

forvalues i=2/47 {

replace tag1=`i' if tag1==. & tag`i'==1

}

drop tag2- _freq

rename tag1 icustay_seq

g id=subject_id*100+icustay_seq

save antipysep_data07b_microbiology.dta, replace

// match microbiological data on icustay id

use "D:\medicine\paper 2014\sepsis and antipyretics\data process\icustay_event.dta", clear

keep subject_id icustay_id icustay_seq

drop icustay_seq

sort subject_id icustay_id

by subject_id, sort : egen float icustay_seq = seq()

g id=subject_id*100+icustay_seq

merge 1:1 id using "D:\medicine\paper 2014\sepsis and antipyretics\data process\antipysep_data07b_microbiology.dta", keep(match) nogenerate

drop subject_id icustay_seq id

g micro_tag=1

save antipysep_data07_microbiology.dta, replace

log close

exit
